# STMN1 expression is associated with chemotherapy and immunotherapy efficacy in biliary tract cancer

**DOI:** 10.3389/fonc.2026.1859927

**Published:** 2026-07-20

**Authors:** Changcheng Wang, Lingxi Nan, Qingyang Meng, Chengjia Lu, Wenkai Cui, Tao Suo, Shulong Zhang, Houbao Liu, Yueqi Wang, Wenqing Qiu, Xiaobo Bo

**Affiliations:** 1 Department of Biliary Surgery, Zhongshan Hospital, Fudan University, Shanghai, China; 2 Biliary Tract Diseases Institute, Fudan University, Shanghai, China; 3 Cancer Center, Zhongshan Hospital, Fudan University, Shanghai, China; 4 Department of General Surgery, Central Laboratory, Shanghai Xuhui Central Hospital, Fudan University, Shanghai, China

**Keywords:** biliary tract cancer, immunotherapy, prognosis, STMN1, tumor immune

## Abstract

**Introduction:**

The prognostic role of STMN1 and its regulatory effects on the tumor microenvironment (TME) in biliary tract cancer (BTC) remain poorly defined. This study aimed to investigate the prognostic value of STMN1 and its predictive capacity for chemotherapy and immunotherapy responses among patients with BTC.

**Methods:**

Six independent cohorts encompassing tissue microarray specimens and transcriptional profiling data from BTC patients were included in our analysis. STMN1 protein expression was quantified via immunohistochemistry (IHC) on tissue microarrays.

**Results:**

STMN1 expression was significantly correlated with inferior overall survival (OS). Multivariate regression further identified STMN1 as an independent adverse prognostic biomarker for OS. Gene Ontology (GO) and Gene Set Enrichment Analysis (GSEA) revealed that elevated STMN1 expression was tightly linked to immune response activation and T-cell infiltration, enabling the formation of an immune-inflamed TME in BTC. IHC validation confirmed positive correlations between STMN1 levels and anti-tumor CD8⁺ T-cell infiltration as well as programmed death-ligand 1 (PD-L1) expression. Patients with low STMN1 expression derived prominent survival benefits from gemcitabine-based adjuvant chemotherapy (ACT), whereas high STMN1 expression predicted superior therapeutic responses to immune checkpoint blockade (ICB) in BTC recipients.

**Discussion:**

STMN1 functions as an independent prognostic biomarker and differential predictive indicator for ACT and ICB efficacy in BTC, likely facilitating the construction of an immune-inflamed TME by promoting tumor immune cell recruitment and infiltration.

## Introduction

Biliary tract cancer (BTC), consisting of intrahepatic cholangiocarcinoma, extrahepatic cholangiocarcinoma, and gallbladder cancer, ranks among the most fatal human malignancies, with an unfavorable overall prognosis and a 5-year survival rate of merely 5%–15% (1). BTC incidence exhibits prominent geographic disparities and has risen gradually across China over recent decades (2, 3). Characterized by a high malignancy potential, BTC readily undergoes early local invasion and distant metastasis (4). According to the latest NCCN Clinical Practice Guidelines, gemcitabine-based chemotherapy constitutes the standard first-line regimen for patients with locally advanced BTC (5). Nevertheless, the clinical benefits derived from conventional chemotherapy and targeted therapies remain suboptimal, substantially compromising long-term patient prognosis. Immunotherapy has achieved durable clinical efficacy across multiple solid tumor types in recent years, yet only a minority of BTC patients obtain objective therapeutic responses (6). Accumulating molecular evidence indicates that oncogenic pathway activation within malignant cells suppresses the initiation and execution of endogenous anti-tumor immune responses by disrupting CD8^+^ T-cell tumor infiltration (7). Accordingly, elucidating how oncogenic signaling drives anti-tumor immune evasion is critical for understanding the molecular basis of treatment resistance in BTC.

In a previous study conducted at our institute, we confirmed that downregulation of STMN1 expression in gallbladder cancer cells can promote the apoptosis of tumor cells and inhibit the proliferation and invasion of biliary tract cancer cells (8, 9). Furthermore, we explored the prognostic value of STMN1 expression in 70 cases of gallbladder cancer and identified STMN1 expression as an independent prognostic factor for overall survival in these patients (10). Moreover, our previous RNA-sequencing analysis on paired gallbladder cancer (GBC) and peritumoral normal tissues (n = 20) demonstrated that circAATF correlates positively with intratumoral CD4^+^ T-cell abundance and PD-L1 expression; in humanized GBC xenograft models, circAATF upregulation potentiates the anti-tumor efficacy of PD-L1 blockade therapy (11). Building upon these preliminary findings, the present study aimed to comprehensively investigate the association between STMN1 expression and gemcitabine-based chemotherapy responsiveness in expanded clinical cohorts, while also exploring STMN1-mediated remodeling of the BTC immune microenvironment.

## Patients and methods

### Patients

Six independent clinical and public datasets were enrolled in this study.

ZS-BTC cohort: A total of 208 consecutive BTC patients who underwent curative surgical resection between January 2009 and October 2013 at Zhongshan Hospital, Fudan University (Shanghai, China) were retrospectively recruited. Among these patients, 71 received at least one cycle of adjuvant gemcitabine-based chemotherapy after surgery. The study protocol was approved by the Ethics Committee of Zhongshan Hospital (approval No. B2014-029), and written informed consent was obtained from all enrolled participants.

ZS-TIB cohort: This cohort comprised 15 patients enrolled in a prospective, open-label, single-arm, single-center phase II clinical trial (NCT03796429) aiming to evaluate the efficacy and safety of toripalimab plus gemcitabine/s-1 (GS) combination chemotherapy for advanced BTC (12). Patient recruitment spanned January 2019 to August 2020 at Zhongshan Hospital, with ethical approval granted by the Institutional Ethics Committee (B2018-294). All trial procedures complied with the Declaration of Helsinki and international Good Clinical Practice guidelines.

Baseline clinicopathological parameters, including age, sex, tumor differentiation status, vascular invasion, and tumor–node–metastasis (TNM) stage, were retrospectively collected for all patients. Tumor TNM staging was independently reviewed and confirmed by two experienced pathologists from the Department of Pathology, Zhongshan Hospital. OS was defined as the interval from curative surgery to all-cause mortality or last clinical follow-up for censored cases. All patients completed follow-up ranging from 2 months to 120 months.

Four additional publicly available transcriptomic cohorts were retrieved for integrated bioinformatic analysis: 118 cholangiocarcinoma (CCA) samples (GSE89749) (13), 217 CCA transcriptional data from OEP002768 (14), 20 gallbladder cancer (GBC) transcriptional data from GSE138109 (11), and 36 CCA transcriptional data from TCGA-CHOL.

### Tissue microarray and immunohistochemical staining

Formalin-fixed paraffin-embedded (FFPE) tumor tissues were used to construct tissue microarrays (TMAs), followed by immunohistochemistry (IHC) staining as previously described. Primary antibodies were used at optimized dilutions as follows: anti-STMN1 (1:600; Cell Signaling Technology, MA, USA), anti-CD8 (ready-to-use, IR623; DAKO, Denmark), anti-FOXP3 (1:200; ab22510, Abcam, USA), and anti-PD-L1 (1:1000; ab23726, Abcam, USA). Positive immunostaining was defined as membranous and/or cytoplasmic staining in malignant epithelial cells. Two independent pathologists, blinded to clinical data, scored IHC staining using a semi-quantitative immunoreactive scoring system. The optimal cutoff value stratifying patients into STMN1-high and STMN1-low subgroups was calculated using the X-tile software.

### Bioinformatics analysis

Transcriptomic data of BTC from TCGA-CHOL, GEO (GSE89749, GSE138109), and National Omics Data Encyclopedia (OEP002768) were extracted to quantify STMN1 expression. The median STMN1 expression value was set as the cutoff to dichotomize samples into STMN1-high and STMN1-low subgroups. We subsequently assessed the prognostic impact of STMN1 and its correlation with tumor-infiltrating immune cells. Single-sample gene set enrichment analysis (ssGSEA) and the TIMER algorithm embedded in the IOBR R package were used to quantify the abundance of diverse intratumoral immune cell subsets.

### Statistical analysis

All statistical analyses were performed using SPSS 22.0 (SPSS Inc., Chicago, IL, USA) and MedCalc v15.2.2 (MedCalc, Mariakerke, Belgium). Categorical variables were compared via the χ² test or Mann–Whitney U test, whereas continuous variables were analyzed with Student’s t-test. Kaplan–Meier survival curves and log-rank tests were used to compare OS between STMN1 subgroups. Univariate and multivariate Cox proportional hazards regression models were constructed to screen independent prognostic determinants. Time-dependent receiver operating characteristic (ROC) curves were generated by integrating STMN1 expression weights and conventional TNM staging to improve prognostic stratification. All statistical tests were two-tailed, and P < 0.05 was defined as statistically significant.

## Results

### Prognostic value of STMN1 expression in biliary tract cancer

**Figure 1:**
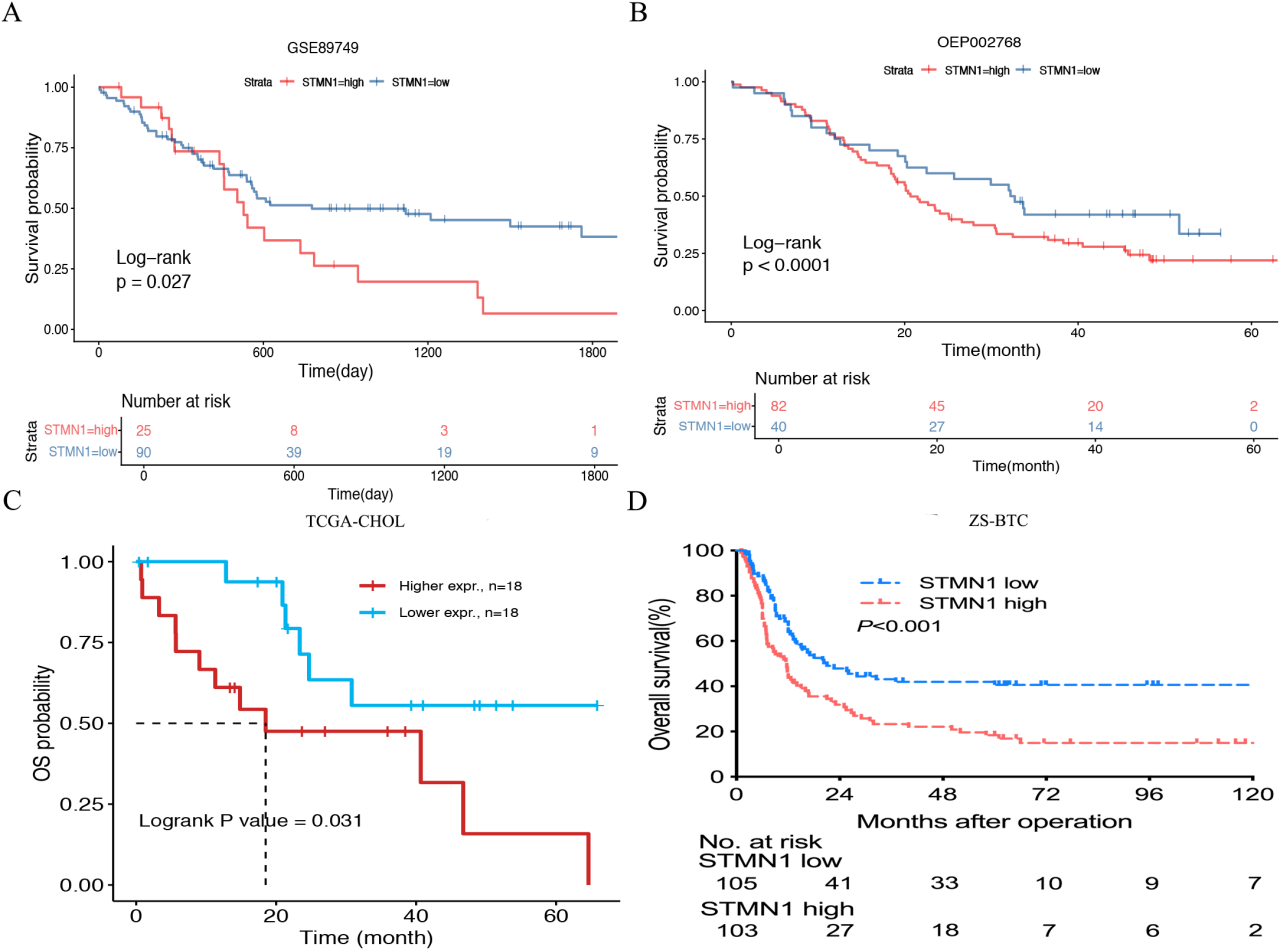
STMN1 yields a poor prognosis in biliary tract cancer patients. **(A)** Kaplan–Meier analysis of OS in BTC patients from the GSE89749 cohort. **(B)** Kaplan–Meier analysis of OS in BTC patients from the OEP002768 cohort. **(C)** Kaplan–Meier analysis of OS in BTC patients from the TCGA-CHOL cohort. **(D)** Kaplan–Meier analysis of OS in BTC patients from the ZS-BTC cohort.

**Figure 2:**
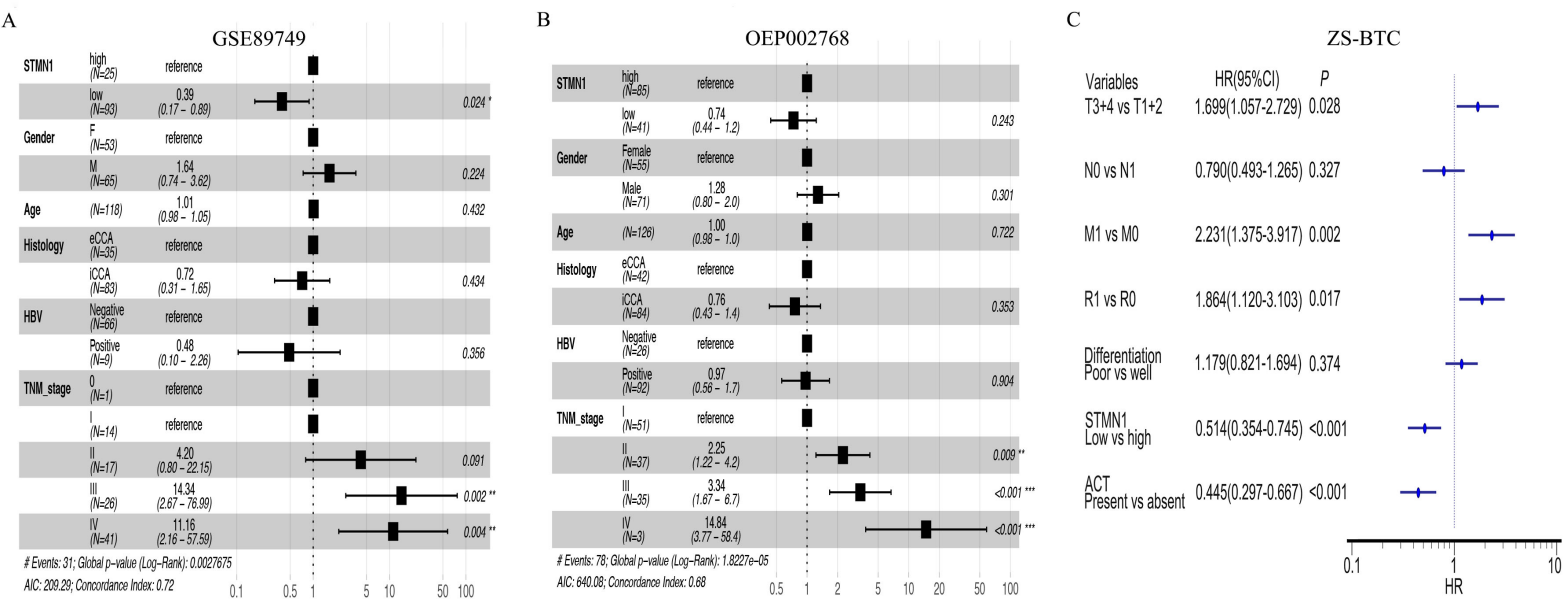
Multivariate Cox regression analysis of OS according to STMN1 expression in patients with biliary tract cancer. **(A)** GSE89749 cohort; **(B)** OEP002768 cohort; and **(C)** ZS-BTC cohort.

Correlations between STMN1 expression and patient clinicopathological characteristics are summarized in **Tables 1**–**3**. As shown in **Table 3**, elevated STMN1 expression in BTC tumor tissues was positively correlated with poor histological differentiation (P < 0.001) and vascular invasion (P = 0.01), whereas no significant associations were observed for other clinical covariates. Kaplan–Meier survival analyses across four independent cohorts (GSE89749, OEP002768, TCGA-CHOL, and ZS-BTC) consistently demonstrated that patients with high tumoral STMN1 expression exhibited significantly poorer OS than those with low STMN1 expression (**Figure 1A**, P = 0.027; **Figure 1B**, P < 0.001; **Figure 1C**, P = 0.031; **Figure 1D**, P < 0.001). Multivariate Cox regression further validated STMN1 as an independent unfavorable prognostic biomarker for OS in both the GSE89749 and ZS-BTC cohorts (**Figure 2A**, P = 0.024; **Figure 2C**, P < 0.001).

**Table 1:** Clinicopathological characteristics of patients with biliary tract cancer in the GSE89749 cohort.

	High	Low	P-value
	(N = 25)	(N = 93)	
Sex			
Female	12 (48.0%)	41 (44.1%)	0.902
Male	13 (52.0%)	52 (55.9%)	
Age			
Mean (SD)	60.0 (12.2)	58.0 (11.8)	0.466
Median [Min, Max]	60.0 [26.0, 79.0]	57.0 [29.0, 78.0]	
Missing	0 (0%)	1 (1.1%)	
Histology			
eCCA	8 (32.0%)	27 (29.0%)	0.967
iCCA	17 (68.0%)	66 (71.0%)	
HBV			
Negative	16 (64.0%)	50 (53.8%)	0.647
Positive	1 (4.0%)	8 (8.6%)	
Missing	8 (32.0%)	35 (37.6%)	
T_stage			
T1	4 (16.0%)	17 (18.3%)	0.937
T2	4 (16.0%)	18 (19.4%)	
T3	12 (48.0%)	38 (40.9%)	
T4	3 (12.0%)	14 (15.1%)	
Tis	0 (0%)	1 (1.1%)	
Missing	2 (8.0%)	5 (5.4%)	
N_stage			
N0	15 (60.0%)	47 (50.5%)	0.626
N1	6 (24.0%)	29 (31.2%)	
N2	0 (0%)	1 (1.1%)	
Missing	4 (16.0%)	16 (17.2%)	
M_stage			
M0	21 (84.0%)	79 (84.9%)	1
M1	2 (8.0%)	7 (7.5%)	
Missing	2 (8.0%)	7 (7.5%)	
TNM_stage			
I	3 (12.0%)	11 (11.8%)	0.943
II	4 (16.0%)	13 (14.0%)	
III	6 (24.0%)	20 (21.5%)	
IV	7 (28.0%)	34 (36.6%)	
0	0 (0%)	1 (1.1%)	
Missing	5 (20.0%)	14 (15.1%)	

**Table 2:** Clinicopathological factors of Biliary Tract Cancer Patients in GSE138109 cohort.

	High	Low	P-value
	(N = 85)	(N = 41)	
Sex			
Female	35 (41.2%)	20 (48.8%)	0.539
Male	50 (58.8%)	21 (51.2%)	
Age			
Mean (SD)	60.5 (11.9)	62.7 (10.2)	0.28
Median [Min, Max]	61.0 [27.0, 86.0]	63.0 [37.0, 85.0]	
Histology			
eCCA	33 (38.8%)	9 (22.0%)	0.0928
iCCA	52 (61.2%)	32 (78.0%)	
HBV			
Negative	18 (21.2%)	8 (19.5%)	1
Positive	62 (72.9%)	30 (73.2%)	
Missing	5 (5.9%)	3 (7.3%)	
T_stage			
T1	37 (43.5%)	18 (43.9%)	0.13
T2	29 (34.1%)	15 (36.6%)	
T3	12 (14.1%)	1 (2.4%)	
T4	7 (8.2%)	7 (17.1%)	
N_stage			
N0	72 (84.7%)	39 (95.1%)	0.229
N1	12 (14.1%)	2 (4.9%)	
N2	1 (1.2%)	0 (0%)	
M_stage			
M0	83 (97.6%)	40 (97.6%)	1
M1	2 (2.4%)	1 (2.4%)	
TNM_stage			
I	34 (40.0%)	17 (41.5%)	0.944
II	24 (28.2%)	13 (31.7%)	
III	25 (29.4%)	10 (24.4%)	
IV	2 (2.4%)	1 (2.4%)	

**Table 3:** Demographics and clinicopathological characteristics of patients in the ZS-BTC cohort.

Characteristic	STMN1 low (n = 105)	STMN1 high (n = 103)	P-value
Age at surgery, years^a^			0.18
Mean ± SD	62.78 ± 11.45	64.85 ± 10.97	
Sex			0.44
Female	66	70	
Male	39	33	
T stage			0.68
T1-2	49	51	
T3-4	56	52	
N stage			0.78
N0	54	55	
N1	51	48	
TNM stage			0.91
I-II	37	41	
III-IV	68	62	
Tumor location			
Gallbladder	69	71	
Perihilar and Distal	36	32	
Tumor differentiation			<0.001
Well/moderate	69	28	
Poor	36	75	
Residual tumor			0.06
R0	88	95	
R1	17	8	
Vascular invasion			0.01
Absent	77	58	
Present	28	45	
ACT			0.23
Absent	65	72	
Present	40	31	

SD, standard deviation; ACT, adjuvant chemotherapy.

^a^The results of continuous variables are presented as mean ± standard deviation (SD). Bold value is considered statistically significant.

### STMN1 fosters an immune-inflamed microenvironment in biliary tract cancer

**Figure 3:**
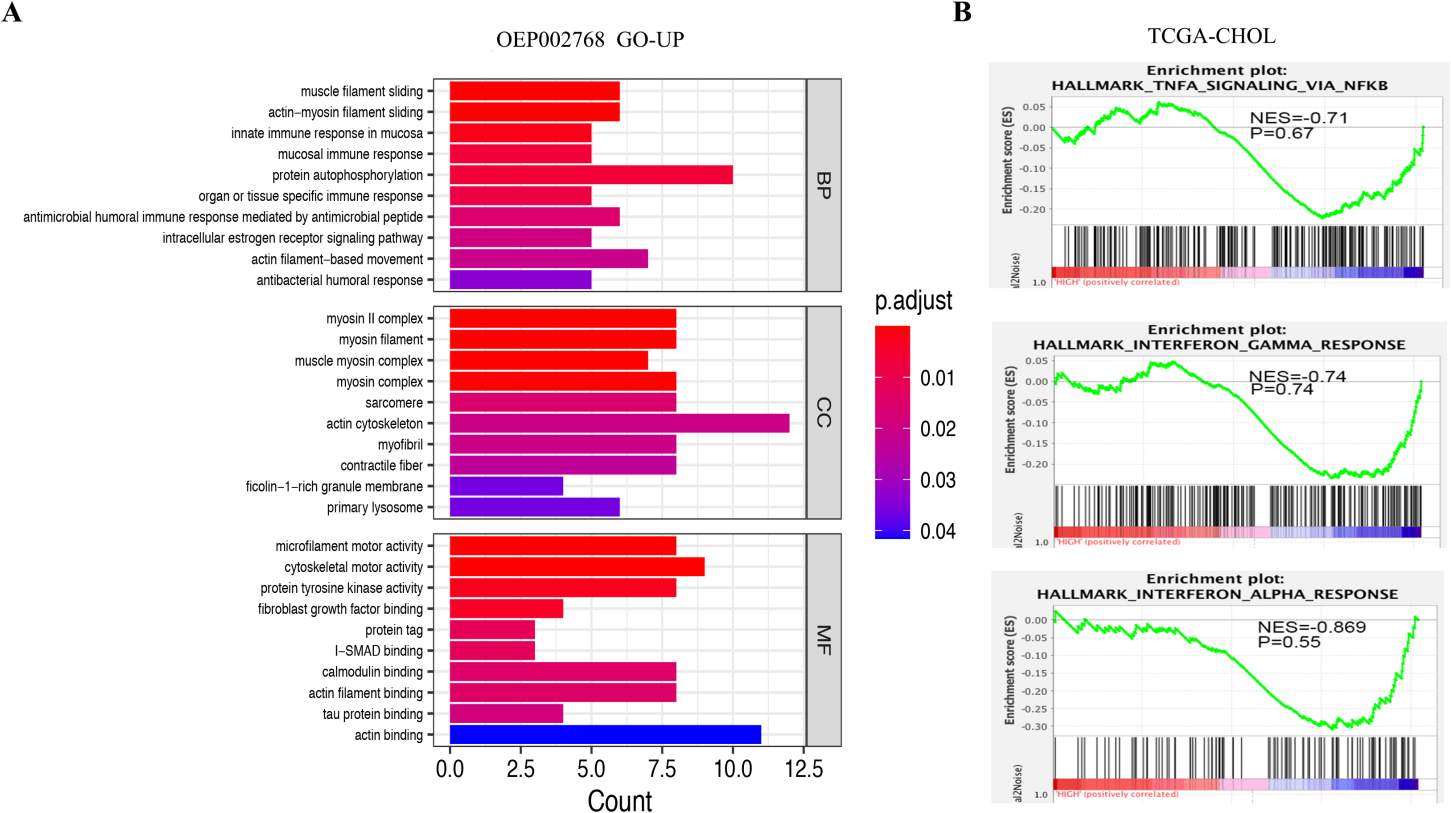
High STMN1 expression defines the immune-inflamed context in BTC. **(A)** Gene Ontology (GO) analysis of the differentially expressed genes (DEGs) in the GSE89749 cohort; **(B)** Gene set enrichment analysis (GSEA) analysis of biological pathways in the TCGA-CHOL cohort.

Previous hepatocellular carcinoma (HCC) studies have linked STMN1 overexpression to the transcription of immune modulators and chemokines. In breast cancer cell lines, STMN1 mRNA levels have also been associated with vascular biomarkers, hypoxia signatures, and PD-L1 transcription. To investigate STMN1-associated signaling pathways in BTC, Gene Ontology (GO) enrichment analysis was performed on differentially expressed genes (DEGs) stratified by STMN1 expression within the GSE89749 cohort. Biological process enrichment revealed that DEGs upregulated in the STMN1-high subgroup were predominantly enriched in innate immune activation, mucosal immunity, and tissue-specific immune responses (**Figure 3A**). Subsequent gene set enrichment analysis (GSEA) further identified multiple T-cell-associated signaling pathways preferentially enriched upon high STMN1 expression, including interferon-γ signaling (NES = 2.46), T-cell-mediated cytotoxicity (NES = 2.36), and leukocyte chemotaxis (NES = 2.24; **Figure 3B**).

**Figure 4:**
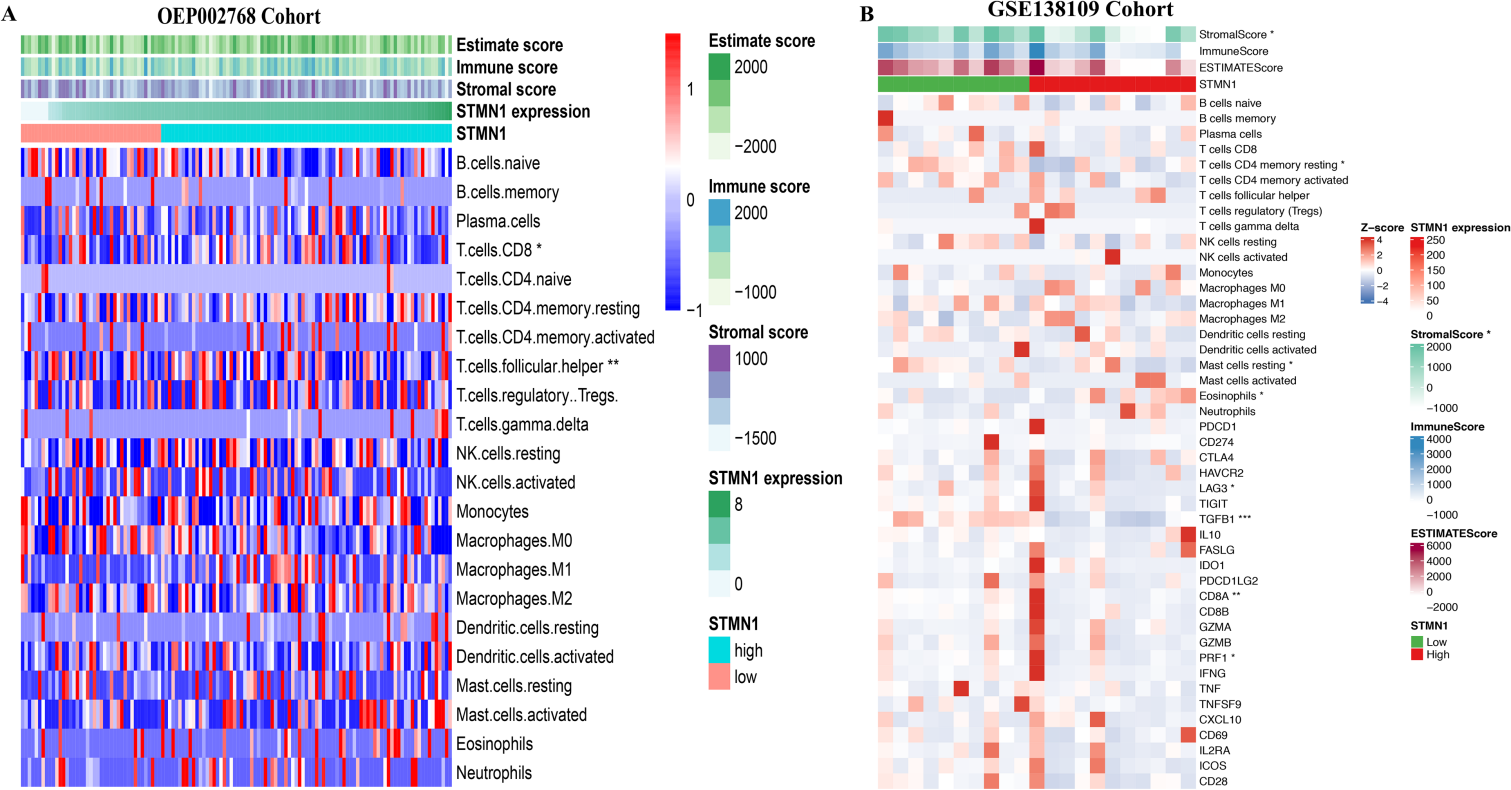
Infiltration of immune cells in BTC stratified by STMN1 expression. **(A)** Heat map showing the comprehensive immune landscape, including ESTIMATE score and 22 types of immune cells, in the OEP002768 cohort; **(B)** Heat map showing the comprehensive immune landscape containing ESTIMATE score, 22 types of immune cells, immune checkpoints, and inhibitory molecules in the GSE138109 cohort.

We next applied the CIBERSORT algorithm to deconvolve the relative fractions of 22 human immune cell subsets in the GSE138109 and OEP002768 cohorts. In the OEP002768 cohort, follicular helper T cells and CD8^+^ T cells were significantly enriched in STMN1-high tumors (**Figure 4A**). In the GSE138109 cohort, elevated STMN1 correlated with increased resting mast cells and memory CD4^+^ T-cell infiltration. Consistently, core effector CD8^+^ T-cell signature genes (CD8A, LAG3, PRF1) displayed positive linear correlations with STMN1 expression across datasets (**Figure 4B**).

### Associations of STMN1 with PD-L1 and tumor-infiltrating immune cells in clinical BTC specimens

**Figure 5:**
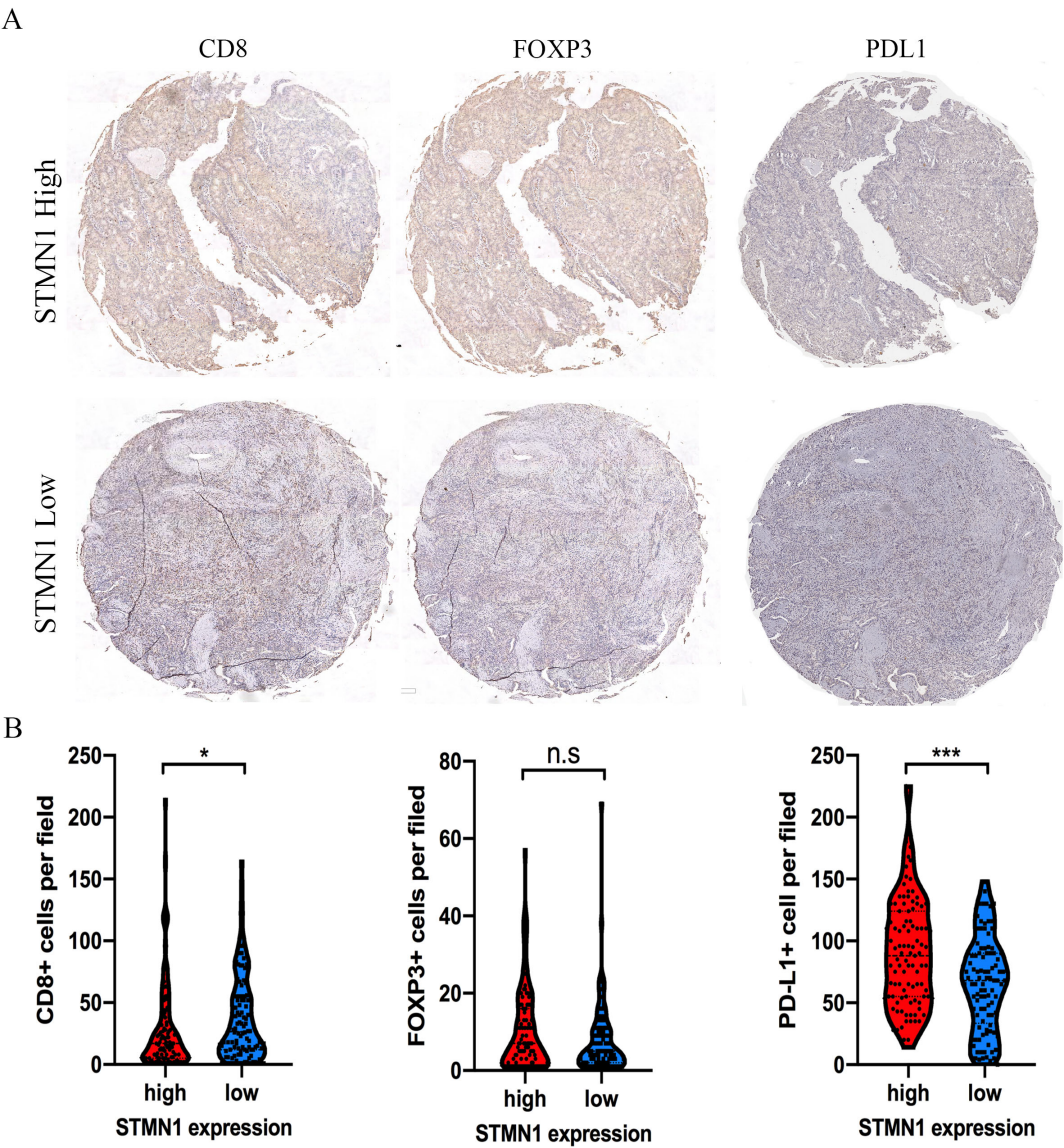
Correlation between STMN1 and CD8^+^ TIL cells, regulatory T cells, and PDL1 in BTC. **(A)** Serial sections from BTC immunohistochemistry stained for CD8, Foxp3, and PDL1. **(B)** Comparison of CD8^+^ T cells, Tregs, and PDL1 level in the STMN1-high and STMN1-low groups in the ZS-BTC cohort *: P<0.05; ***: P<0.001; ns: P>0.05.

Given the robust transcriptomic link between STMN1 and the tumor microenvironment (TME) composition, serial-section IHC staining was conducted to quantify intratumoral PD-L1, CD8^+^ T-cell, and FOXP3^+^ regulatory T-cell densities in the ZS-BTC cohort. Unexpectedly, tumors with low STMN1 expression harbored significantly higher CD8^+^ T-cell infiltration than STMN1-high tumors (**Figure 5B**, P = 0.02). STMN1 protein expression showed a strong positive correlation with tumoral PD-L1 levels (**Figure 5B**, P < 0.001), whereas FOXP3^+^ T-cell infiltration was comparable between STMN1-high and STMN1-low subgroups (**Figure 5B**, P = 0.06). Collectively, these IHC findings suggest that high STMN1 expression contributes to suppressed anti-tumor immune reactivity in BTC, which may partially explain the poorer survival outcomes observed in patients with high STMN1 expression.

### STMN1 differentially predicts clinical responses to ACT and ICB in BTC

**Figure 6:**
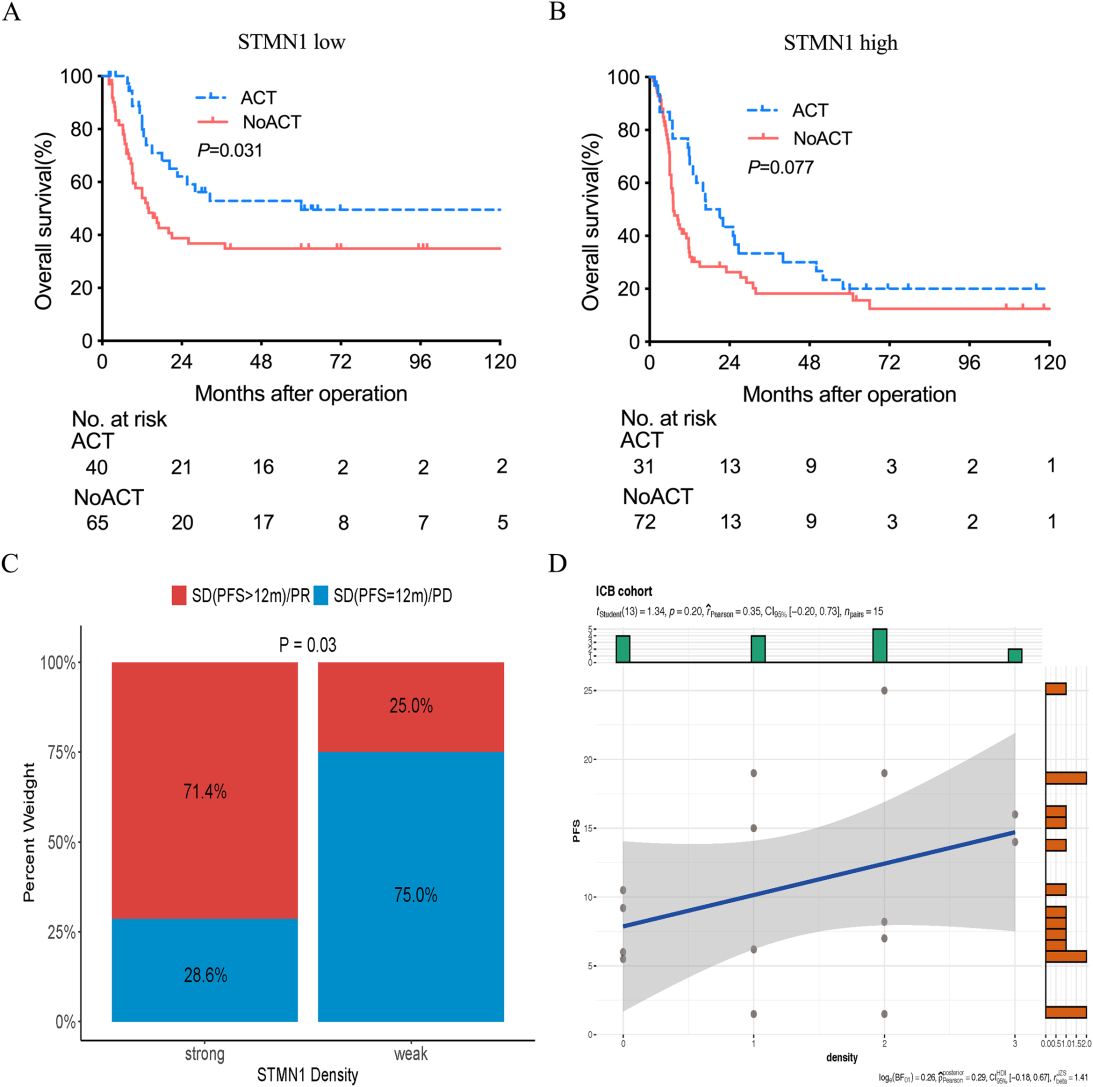
STMN1 expression predicts responsiveness to ACT and ICB in BTC patients. **(A, B)** Kaplan–Meier survival analysis of responsiveness to ACT in STMN1-low and STMN1-high patient subgroups; **(C)** Stacked bar plot demonstrating responsiveness to toripalimab according to STMN1 expression in the ICB cohort; **(D)** Correlations assessed by Pearson analysis between progression-free survival (PFS) and STMN1 expression.

Our previous GBC cohort study identified STMN1 as a candidate predictive marker for adjuvant gemcitabine chemotherapy. Herein, stratification analysis of the ZS-BTC cohort confirmed that patients with low tumoral STMN1 expression derived significant survival benefits from gemcitabine-based adjuvant chemotherapy (ACT), whereas those with high STMN1 expression derived minimal therapeutic gains (P = 0.031 vs P = 0.077; **Figures 6A, B**).

For immune checkpoint blockade (ICB) predictive validation, we analyzed the ZS-TIB cohort treated with toripalimab combined with GS chemotherapy. Patients in the STMN1-high subgroup achieved a significantly higher objective response rate than those in the STMN1-low subgroup (P = 0.03; **Figure 6C**), alongside a trend toward prolonged progression-free survival (PFS; P = 0.20, R = 0.35; **Figure 6D**).

## Discussion

Aberrant STMN1 overexpression is frequently detected across diverse human malignancies and correlates with enhanced oncogenic aggressiveness (15–17). Multiple clinical studies have further demonstrated that elevated STMN1 expression is associated with poor patient prognosis (18–21). In the present study, we verified that increased tumoral STMN1 expression in BTC correlates with poor tumor differentiation, unfavorable overall survival (OS), and limited responsiveness to gemcitabine-based ACT. Multivariate modeling further confirmed STMN1 as an independent adverse prognostic biomarker. Moreover, the integration of STMN1 expression with conventional TNM staging substantially improved the precision of BTC prognostic stratification.

The majority of BTC patients are diagnosed at an advanced disease stage, leaving systemic chemotherapy as the primary therapeutic option for improving clinical outcomes (22, 23). As one of the most effective cytotoxic agents for BTC management, gemcitabine-based regimens are widely recommended as first-line standard care for advanced BTC (22, 24, 25). Therefore, reliable predictive biomarkers are urgently needed to screen patients who are most likely to benefit from adjuvant gemcitabine chemotherapy. Previous translational investigations have established causal links between STMN1 upregulation and paclitaxel chemoresistance in breast, lung, and ovarian cancers (15, 16, 26–29). Consistently, a gastric cancer study reported that STMN1 overexpression predicts poor paclitaxel responsiveness, whereas STMN1 knockdown restores tumor cell chemosensitivity to paclitaxel (20). Our BTC clinical data extend these findings by demonstrating that high STMN1 abrogates survival benefits from gemcitabine-based ACT, highlighting STMN1 as a feasible predictive marker for gemcitabine efficacy in BTC.

Classically, STMN1 drives malignant progression by modulating microtubule assembly to alter tumor cell proliferation, invasion, and apoptotic susceptibility (30). Emerging evidence underscores that tumor progression relies not only on intrinsic oncogenic mutations but also on dynamic immune crosstalk within the tumor microenvironment (TME) (6), with constitutive oncogenic signaling frequently crippling endogenous anti-tumor immune surveillance (31). Therefore, elucidating the molecular mechanisms underlying TME remodeling may facilitate the development of novel therapeutic strategies and optimize immunotherapeutic efficacy in BTC. Previous studies in head and neck squamous cell carcinoma (HNSCC) reported positive correlations between STMN1 expression and multiple immune checkpoint molecules, including PD-L1, TIM3, and B7-H3 (32). HCC-based transcriptomic analyses further demonstrated associations between elevated STMN1 expression and increased infiltration of CD4^+^ T cells, CD8^+^ T cells, B cells, and myeloid immune subsets (33). Combining multi-cohort transcriptomics and TMA-based IHC validation, our study corroborated that STMN1 upregulation correlates with comprehensive intratumoral immune cell infiltration in BTC, reinforcing the central role of immune microenvironment remodeling during BTC initiation and progression.

Consistent with previous HCC research reporting STMN1-mediated transcriptional regulation of immune modulators and TME immune composition, our GO and GSEA analyses identified significant enrichment of interferon-γ and cytotoxic T-cell effector pathways in STMN1-high BTC tumors, phenotypically defining an immune-inflamed TME subtype favorable to ICB treatment. Consistent with this observation, our prospective ICB cohort data demonstrated intensified tumoral STMN1 expression in toripalimab combination responders relative to non-responders, supporting the clinical utility of STMN1 as a predictive stratification biomarker for anti-PD-1 immunotherapy in BTC.

Several limitations should be acknowledged. First, all retrospective analyses are susceptible to inherent selection bias, and the ZS-TIB immunotherapy cohort included a limited number of enrolled patients. Second, although our clinical data support robust prognostic and predictive performance of STMN1, the exact molecular cascades through which STMN1 modulates CD8^+^ tumor-infiltrating lymphocyte recruitment remain unclear and warrant further mechanistic investigation. Finally, large-scale prospective external validation cohorts are needed before STMN1-based patient stratification can be implemented in routine clinical practice.

In conclusion, our multi-cohort study validates STMN1 as an independent adverse prognostic biomarker in BTC. Low tumoral STMN1 expression identifies patients who are most likely to gain clinical benefits from gemcitabine-based adjuvant chemotherapy, whereas high STMN1 expression drives the formation of an immune-inflamed TME and may serve as a clinically actionable predictive biomarker for improved anti-PD-1 immunotherapy responsiveness in BTC.

## Data Availability

The raw sequencing reads have been deposited in the Gene Expression Omnibus (GEO, http://www.ncbi.nlm.nih.gov/geo/) database, under the accession code GSE138109. The Gene expression data have been deposited at the Gene Expression Omnibus (GEO; www.ncbi.nlm.nih.gov/geo) with GEO accession numbers GSE89749. The RNA‐seq data can be viewed in the National Omics Data Encyclopedia (NODE database: OEP002768). 36 cases of cholangiocarcinoma from TCGA were analyzed, and mRNA expression data were downloaded from cBioPortal in the RSEM format.
