# Supplementary figures and images for: STMN1 expression is associated with chemotherapy and immunotherapy efficacy in biliary tract cancer
Source: Front Oncol. 2026 Jul 20;16:1859927. doi: 10.3389/fonc.2026.1859927 (PMC13429508; doi:10.3389/fonc.2026.1859927)

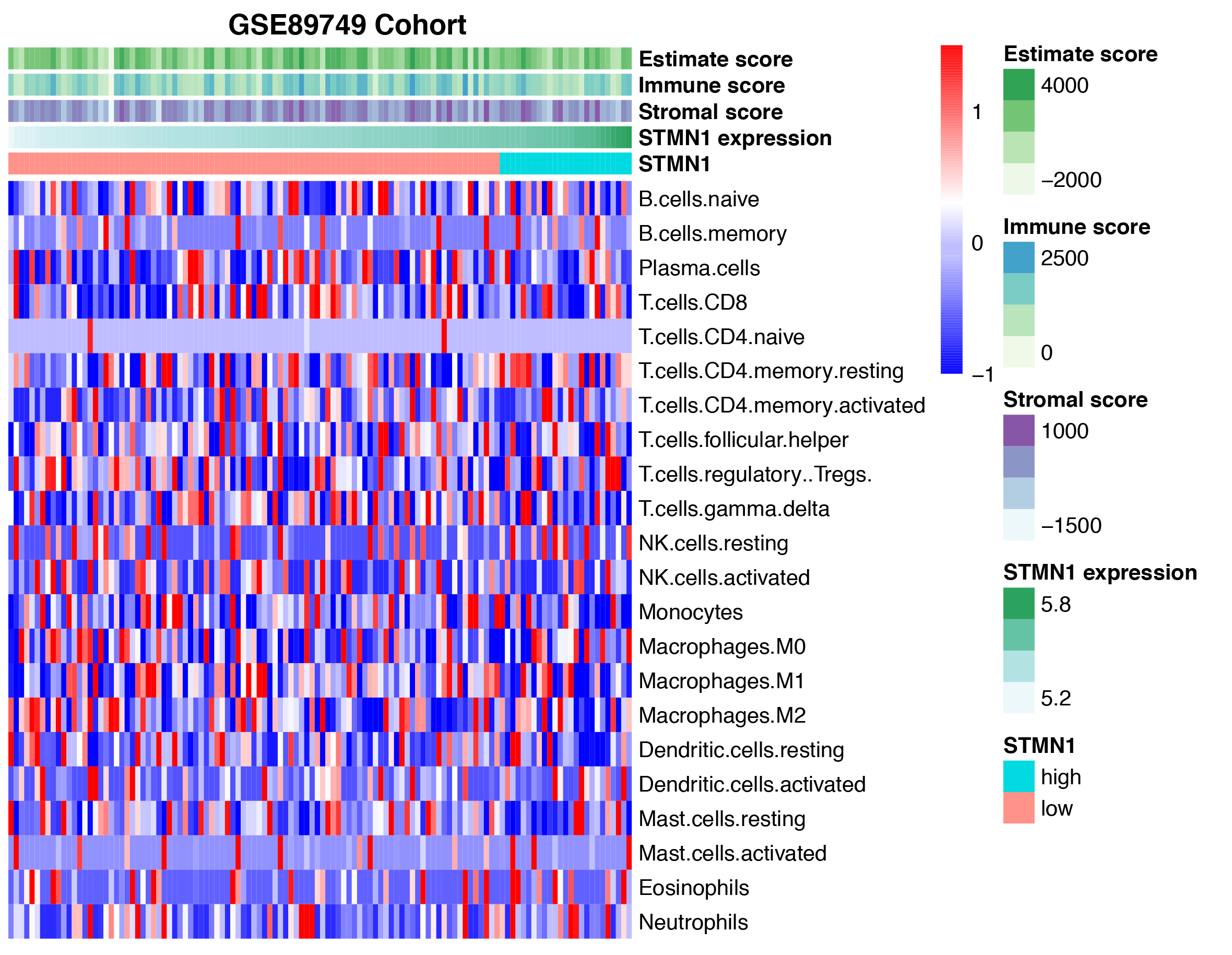

Supplement: Supplementary file 1 [file Image1.tif]

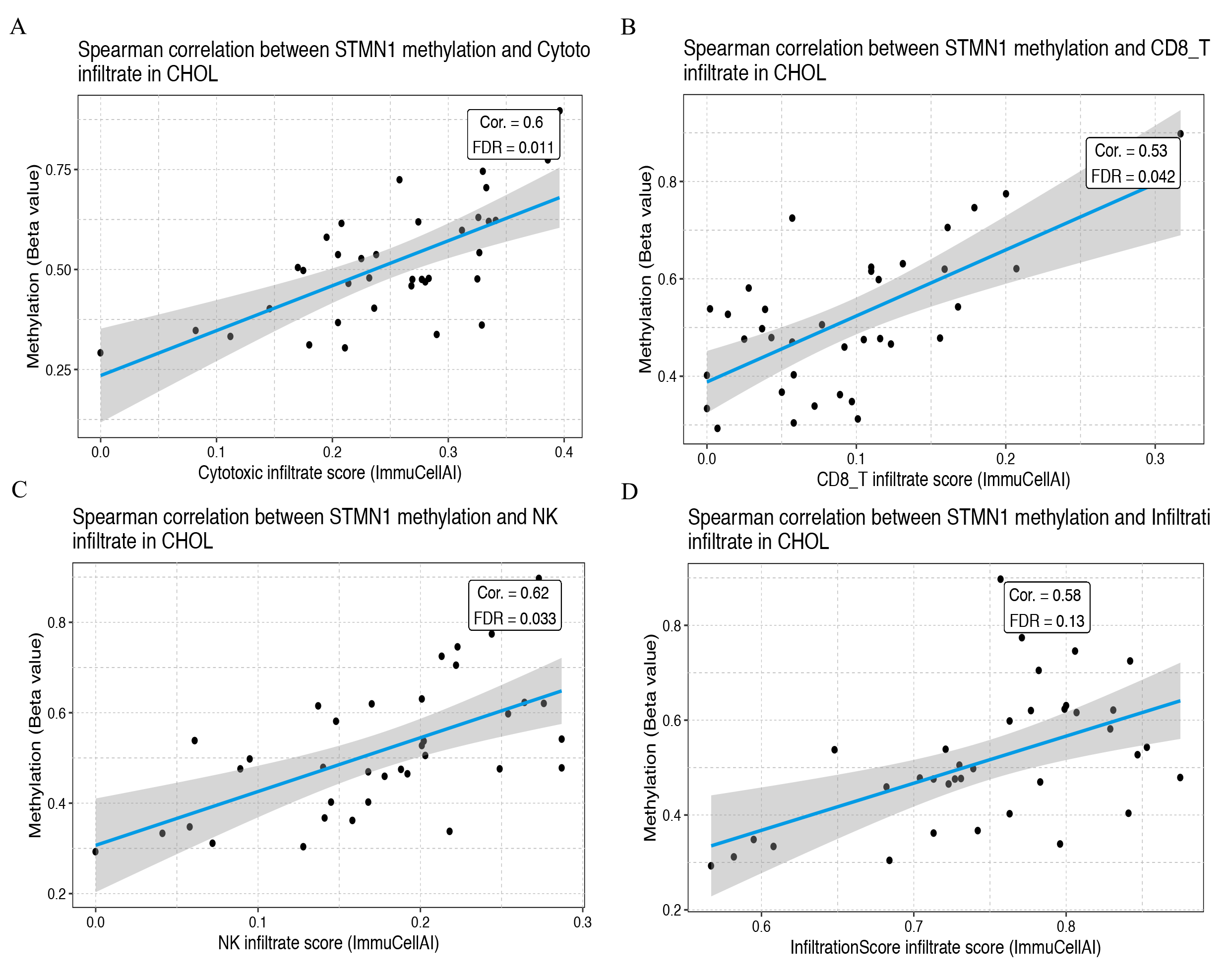

Supplement: Supplementary file 2 [file Image2.tif]
